# Interpretable machine-learning model to accurately identify women at risk of excessive gestational weight gain

**DOI:** 10.3389/fpubh.2026.1779962

**Published:** 2026-04-20

**Authors:** Linyan He, Xihong Zhou, Yuehan Shen, Bing Zhou, Jingni Wu, Jinyu Liu

**Affiliations:** 1 Clinical Nursing Teaching and Research Section, The Second Xiangya Hospital, Central South University, Changsha, Hunan, China; 2 Department of Clinical Laboratory, The Second Xiangya Hospital, Central South University, Changsha, Hunan, China; 3 Department of Obstetrics, The Second Xiangya Hospital, Central South University, Changsha, Hunan, China

**Keywords:** excessive gestational weight gain, interpretable, machine learning, maternal health, nomogram, predictive model

## Abstract

**Objective:**

To establish and validate interpretable machine-learning (ML) models for early assessment and identification of Chinese women at risk of excessive gestational weight gain (EGWG).

**Methods:**

We performed a prospective observational study with pregnant women whose gestational age averaged 19 weeks or less. These women attended the obstetric clinic of a tertiary hospital in CentralSouth China between January and June 2023, and again from April to May 2024. Women completed standardized questionnaires, and their gestational weight gain (GWG) was recorded until delivery. We conducted feature selection by applying the Boruta algorithm together with the least absolute shrinkage and selection operator (LASSO) algorithm, We used four ML models—the logistic regression(LR), light gradient boosting machine(LightGBM), extreme gradient boosting(XGBoost), and random forest (RF) models—optimizing its hyperparameters by grid search and 5-fold crossvalidation. Model performance was evaluated using the area under the receiver operating characteristic curve (AUC), confusion matrix, kappa statistic, calibration curves, and decision curve analysis. To enhance model interpretability, the SHapley Additive exPlanations (SHAP) framework was applied to quantify and rank the contribution of individual predictors. Based on the key predictive features, a web-based interactive calculator was developed using Python and the Flask micro-framework to facilitate clinical application.

**Results:**

We enrolled 578 pregnant women in all. The combined use of the Boruta and LASSO algorithms screened ten critical predictors. The LightGBM model showed superior predictive performance with an accuracy of 88.6%, sensitivity of 87.5%, specificity of 89.9%, kappa statistic of 0.770, and AUC of 0.926 (95% CI: 0.889–0.962) in the test cohort. The SHAP analysis indicated that the body image in pregnancy, protective motivation for gestational weight management, parity, the weekly frequency of consuming sugar-sweetened beverages, desserts, and Western-style fast food, and moderate-intensity physical activity time were the major determinants that influenced model prediction. An online calculator was developed and made available for clinicians at: http://39.103.64.176/.

**Conclusions:**

We established an interpretable ML model for predicting the risk of EGWG. The LGBM model exhibited higher predictive accuracy and may serves as a powerful tool for the early detection and individualized management of the EGWG risk among Chinese pregnant women.

## 1 Introduction

A mother's gestational weight gain (GWG)—that is, her weight change from conception to delivery—indicates their maternal nutritional status and can be linked to negative outcomes in both the mothers and newborns (1, 2). Excessive GWG (EGWG) has increased worldwide, with the sedentary lifestyle prevalent in modern societies significantly increasing obesity rates among women of reproductive age. The prevalence of EGWG varies between 38.2% and 55.2% in various continents (3). China has the second largest population of obese pregnant women, accounting for nearly one-third of the worldwide incidence (4), and may also be related to increased risk for gestational hypertensive disorders, gestational diabetes, postpartum hemorrhage, pelvic floor dysfunction, venous thrombosis, postpartum depression, lasting maternal obesity, and cardiometabolic conditions (2, 5, 6). The offspring of mothers with EGWG are more likely to develop childhood obesity and long-term metabolic disorders (2). Collectively, these trends demonstrate that EGWG may put a great strain on public health services, urging the early detection of the risks and preventive measures. Early detection of women at risk is essential, for example, to the extent that it allows for early detection so as to permit prompt interventions and avoid adverse maternal and child health outcomes.

Previous prediction tools have explored dietary, psychosocial, or sociodemographic factors independently. Hrolfsdottir et al. (7) created a concise dietary screening tool to detect women at risk by analyzing dietary patterns in early pregnancy. Fealy et al. (8) developed a streamlined psychosocial assessment tool that includes evaluations of body image, weight gain attitudes, and self-efficacy. Similarly, Geyer et al. (9) proposed a model integrating sociodemographic characteristics, smoking behavior, and mental-health indicators. However, EGWG arises from multiple interacting behavioral and psychological factors, limiting the predictive performance of single-domain models. McDonald et al. (10) developed a logistic regression model incorporating psychosocial variables, which demonstrated only moderate accuracy (area under curve(AUC): 0.62–0.76), indicating a need for more robust approaches that account for multilevel determinants. Our previous logistic regression analysis revealed that behavioral and psychosocial factors, such as pre-pregnancy overweight or obesity, screen-time eating habits, the weekly frequency of consuming sugar-sweetened beverages, desserts, and Western-style fast food, maternal body image, parity, protective motivation for gestational weight management, and daily moderate-intensity physical activity, are predictors of EGWG, demonstrating strong discrimination in internal validation (11). However, traditional regression may not fully capture non-linear interactions. ML can manage non-linear interactions and complex variable relationships, outperforming traditional regression by improving prediction accuracy and robustness and flexibility (12).

In this research, we developed and externally validated four machine learning models–the logistic regression (LR), light gradient boosting machine (LGBM), extreme gradient boosting (XGB), and random forest (RF) model for predicting the EGWG, we used the SHapley additive exPlanations (SHAP) method to enhance the interpretability. Our aim is to create a clinically useful, accurate tool for predicting EGWG from ML algorithms which can inform targeted preventative strategies and explain the multiplicity of that can contribute to EGWG.

## 2 Materials and methods

### 2.1 Study design

The investigation was conducted across both outpatient services and inpatient units of a tertiary-level obstetric hospital located in Central South China. Participants were recruited during two independent time periods: January–June 2023 (training cohort) and April–May 2024 (test cohort). The 2024 cohort was used as a temporally distinct test set, as it was collected at the same institution but in a distinct recruitment window. This validation approach was intended to evaluate model reproducibility over time under comparable clinical workflows. Pregnant women completed standardized questionnaires and were followed until delivery to obtain their GWG. The EGWG questionnaire was created following a literature review and refined through two expert panel discussions. The inclusion criteria included (1) participants aged 20 years or older, (2) a gestational age of at least 14 weeks, (3) a viable intrauterine pregnancy, and (4) voluntary participation with informed consent. Exclusion criteria included (1) multiple gestations, (2) psychiatric or cognitive impairments, (3) severe pre-existing medical conditions (such as anemia, diabetes, hypertension, cardiac, hepatic, renal, or thyroid diseases), (4) maternal conditions severely affecting weight gain (such as anorexia, bulimia, or history of bariatric surgery), and (5) an inability to independently complete the questionnaire. Elimination criteria included (6) withdrawal, inability to follow-up, or pregnancy termination, (2) patterned or uniform response selection, and (3) more than one-third of the questions unanswered.

Sample size planning followed the framework of Riley et al. (13) and was performed at the design stage assuming up to 25 candidate predictors before feature selection, at least 250 for the 25 candidate predictors in this study. The datasets were divided in a 7:3 ratio, ensuring a minimum of 107 cases in the test dataset. Data from January to June 2023 constituted the training dataset, whereas data from April to May 2024 comprised the test dataset. EGWG was defined according to the 2022 National Health Commission guidelines for recommended GWG (1), and participants with GWG exceeding the recommended range were classified as having EGWG.

The ethics review committee of the Xiangya School of Nursing, Central South University, approved this research (Approval No. E2023183). Participants gave written informed consent prior to enrollment, and the study was conducted and reported in accordance with the Strengthening the Reporting of Observational Studies in Epidemiology (STROBE) guidelines. The authors are open to sharing the data upon request.

### 2.2 Study variables

The questionnaire comprised the following components:

The demographic and lifestyle factors included maternal age, pre-pregnancy body mass index (BMI), residence, education level, monthly income, marital and employment status, parity, smoking and alcohol history, the weekly consumption frequency of sugar-sweetened beverages (SSBs), desserts, and Western fast food (WFF), habit of eating late night snacks or snacks, habit of eating in front of a screen.

The Pregnancy Physical Activity Questionnaire(PPAQ), originally developed by Chasan-Taber et al. (14) and translated by Zhang et al. (15) (content validity index = 0.940, retest reliability = 0.944), included 31 items, categorized into three activity intensity levels based on metabolic equivalents (METs)—that is, sedentary (< 1.5 METs), low (1.6–2.9 METs), and moderate to vigorous activity (≥3.0 METs). For each activity, the total energy expenditure was calculated by multiplying the energy cost by the duration weight. The 2020 World Health Organization (WHO) guidelines on physical activity and sedentary behavior recommend that pregnant women without contraindications should accumulate at least 150 min of moderate-intensity physical activity per week (16). In this study, engaging in activities with an energy expenditure of 3.0–6.0 METs for more than 2.5 h per week was defined as meeting the physical activity requirements, according to the 2020 WHO guidelines.

The Perceived Stress Scale(PSS), developed by Cohen et al. (17), was used to assess the perceived stress levels, as translated by Yang et al. (18), with higher scores reflecting greater perceived stress (Cronbach's α = 0.734).

The Edinburgh Postnatal Depression Scale (EPDS), developed by Cox et al. (19) and translated by Guo (20), was used to assess depressive symptoms (Cronbach's α = 0.76). Evidence from Park et al. (21) further demonstrated that the EPDS was suitable for identifying depressive symptoms during pregnancy and the postnatal period.

The Protective Motivation for Gestational Weight Management Questionnaire (22), developed by the research team based on the Protection Motivation Theory to evaluate the motivational and self-efficacy factors associated with weight control. Higher scores reflected greater motivation (Cronbach's α = 0.894 and test–retest reliability of 0.947).

The Body Image in Pregnancy Scale (BIPS), created by Watson et al. (23) and translated by Sun (24), was used to evaluate body image disturbance. Higher scores indicated increased body image disturbance (Cronbach's α = 0.861).

The Social Support Rating Scale (SSRS), developed by Xiao (25), was used to assess social support levels (Cronbach's α = 0.825 to 0.896). Higher scores reflected higher level of social support.

### 2.3 Data collection

Data were collected by three trained investigators using standardized procedures, and eligible participants were recruited from outpatient and inpatient obstetric settings and completed on-site self-reports. Each questionnaire was checked immediately after completion for omissions or inconsistencies, which were then corrected in real time. Pre-pregnancy weight was obtained from the first prenatal record; pre-delivery weight was measured in the hospital ward one week before delivery using calibrated electronic scales. Both sites used the same brand and model of electronic weighing scales, which had been calibrated and certified for weight measurement.

### 2.4 Data preprocessing and feature selection

Raw data were screened for outliers, missing values, and invalid entries. Records with more than 10% missing responses were excluded. Missing values were addressed using mean imputation, and the data were standardized via Z-score normalization. Feature selection was performed on 25 candidate variables using the LASSO and Boruta algorithms.

The LASSO algorithm was employed for feature selection and modeling of high-dimensional data. This technique applies an *L1* penalty to regression coefficients, which drives some coefficients to zero, thereby performing feature selection and model simplification. In this study, all variables were included as inputs to the LASSO algorithm, with EGWG as the outcome variable. The optimal penalty parameter (λ) was identified through 10-fold cross-validation. The λ value minimizing the mean squared error (λ_*min*_) was chosen to identify the most relevant features. The Boruta algorithm, a random-forest-based wrapper method, was used to assess the variable importance by comparing each feature with a permuted “shadow” counterpart (26). Iterations were continued up to 500 iterations or until stability was achieved. The variables consistently confirmed by both methods were retained as the final predictors for predictive model construction.

### 2.5 Model development, evaluation, and interpretation

Four models were trained—that is, the LR, RF, LightGBM, and XGBoost models. The model were constructed using the sklearn scientific computing library in Python 3.10.16, with the GridSearchCV toolbox employed to identify optimal parameters and conduct training. The final hyperparameter combination was determined through 5-fold cross-validation. The predictive performance was assessed through the area under the receiver operating characteristic curve (AUC), accuracy, precision, specificity, F1-score, kappa coefficient, calibration curves, decision curve analysis (DCA), and clinical impact curve (CIC) metrics. The SHAP framework was used to evaluate the model interpretability, providing global and local interpretability, identifying the most influential features for the overall model and for individual patient cases to quantify each predictor's contribution. A nomogram was subsequently constructed based on key predictors using R ver. 4.5.2 to facilitate clinical application. To facilitate model application in a clinical setting, we developed a web-based interactive calculator using Python and the Flask micro-framework. When the values of the relevant features are provided, this application returns the probability of EGWG.

### 2.6 Statistical analysis

Data analysis was carried out using Microsoft Excel, IBM SPSS Statistics (ver. 26.0), and Python (ver. 3.10.16) for data organization, statistical testing, and predictive modeling, respectively. For normally distributed continuous variables, the results are were presented as mean ± standard deviation and analyzed using independent *t*-tests.Variables exhibiting non-normal distributions were summarized using the median and interquartile range and compared between groups with the Mann–Whitney U test. Categorical data were described as frequencies with corresponding percentages and were examined using either the chi-square test or Fisher's exact test, as appropriate. LASSO regression was implemented using the *glmnet* package in Python; the Boruta algorithm was implemented via Boruta ver. 0.3.0. Pairwise comparisons of AUCs were performed using DeLong's test to assess differences in model discrimination on the test cohort. The 95% confidence intervals for differences of each AUC were constructed via non-parametric bootstrap resampling with 1,000 iterations. The model interpretability was evaluated using the SHAP package (ver. 0.45.0), and a web-based interactive calculator was developed using Python (ver. 3.14.3) and the Flask micro-framework. Statistical significance was defined as a two-sided *p* value less than 0.05.

## 3 Results

### 3.1 Baseline characteristics

A total of 618 participants were initially recruited; 11 invalid questionnaires were excluded, yielding 607 valid responses. 22 women delivered preterm and 7 did not deliver at the study hospital and thus lacked GWG data, so 578 participants were finally included, of which 403 were used in the training dataset, and 175 in the test dataset. The basic characteristics of the study cohort are shown in Table 1. In the training dataset, the participants were aged 22–43 years, with gestational ages 19 ± 5.06 weeks. In the test dataset, participants were aged 20–42 years, with gestational ages 18 ± 4.96 weeks. The overall incidence of EGWG was 50.17% (training dataset, 48.14%; test dataset: 54.49%). An evaluation of the 25 candidate variables indicated that there were no statistically significant differences between the training set and the test set (*p* > 0.05), demonstrating their homogeneity and comparability and supporting the reliability of the test dataset for external model verification.

**Table 1:** The basic characteristics of the study cohort (*n* = 578).

Variables	Training set (*n* = 403)	Test set (*n* = 175)	Test statistic	*P-*value
Age (years)	33.39 ± 5.01	33.31 ± 5.15	−0.163^b^	0.871
Pre-pregnancy BMI (kg/m^2^)			1.882^a^	0.597
18.5–24.0	164 (40.7%)	77 (44.0%)		
< 18.5	78 (19.4%)	26 (14.9%)		
24.0–28.0	107 (26.6%)	46 (26.3%)		
≥28.0	54 (13.4%)	26 (14.9%)		
Level of education			2.242^a^	0.524
Junior high school or below	56 (13.9%)	31 (17.7%)		
High school or secondary school	116 (28.8%)	42 (24.0%)		
College or bachelor's degree	198 (49.1%)	88 (50.3%)		
Master's degree or above	33 (8.2%)	14 (8.0%)		
Marital status			3.796^a^	0.284
Unmarried	76 (18.9%)	43 (24.6%)		
Married	262 (65.0%)	112 (64.0%)		
Divorce/widowed/separation	65 (16.1%)	20 (11.4%)		
Personality			0.471^a^	0.493
Extraversion	200 (49.6%)	93 (53.1%)		
Introversion	203 (50.4%)	82 (46.9%)		
Place of residence			0^a^	1
Towns	310 (76.9%)	134 (76.6%)		
Rural	93 (23.1%)	41 (23.4%)		
Status of employment			2.767^a^	0.096
No	71 (17.6%)	42 (24.0%)		
Yes	332 (82.4%)	133 (76.0%)		
Monthly income (RMB)			7.340^a^	0.062
≤ 4,000	43 (10.7%)	29 (16.6%)		
4,001–8,000	117 (29.0%)	37 (21.1%)		
8,001–15,000	175 (43.4%)	84 (48.0%)		
>15,000	68 (16.9%)	25 (14.3%)		
Parity (time)			3.934^a^	0.14
0	164 (40.7%)	86 (49.1%)		
1	114 (28.3%)	46 (26.3%)		
≥2	125 (31.0%)	43 (24.6%)		
Eating in front of screen			0.046^a^	0.830
No	199 (49.4%)	84 (48.0%)		
Yes	204 (50.6%)	91 (52.0%)		
Habit of eating late night snacks			0.047^a^	0.828
No	227 (56.3%)	101 (57.7%)		
Yes	176 (43.7%)	74 (42.3%)		
Weekly frequency of SSB/Desserts/WFF (time per week)			2.635^a^	0.451
< 1	109 (27.0%)	48 (27.4%)		
1–2	110 (27.3%)	40 (22.9%)		
3–4	100 (24.8%)	41 (23.4%)		
≥5	84 (20.8%)	46 (26.3%)		
Smoking			0.298^a^	0.585
No	394 (97.8%)	169 (96.6%)		
Yes	9 (2.2%)	6 (3.4%)		
Alcohol consumption			0.265^a^	0.607
No	400 (99.3%)	175 (100.0%)		
Yes	3 (0.7%)	0 (0.0%)		
EPDS (score)	9.28 ± 4.59	9.33 ± 4.52	0.110^b^	0.912
BIPS (score)	105.56 ± 24.00	109.42 ± 23.40	1.806^b^	0.072
PSS (score)	14.13 ± 4.16	14.37 ± 3.94	0.639^b^	0.523
SSRS (score)	38.55 ± 7.69	37.57 ± 7.27	−1.465^b^	0.144
PMT (score)	115.24 ± 19.95	118.08 ± 18.26	−1.185^b^	0.237
TDEE (MET-h/daily)	31.72 ± 5.32	31.80 ± 5.52	0.170^b^	0.865
Sedentary time (h)	19.91 ± 2.08	19.94 ± 2.12	0.184^b^	0.854
LPA time (h)	3.69 ± 1.98	3.96 ± 1.90	1.552^b^	0.122
MPA time (h)	0.78 ± 0.60	0.79 ± 0.61	0.173^b^	0.863
VPA time (h)	0.01 ± 0.03	0.01 ± 0.03	1.362^b^	0.174
Meeting WHO guidelines for physical activity			0.059^a^	0.808
No	188 (46.7%)	79 (45.1%)		
Yes	215 (53.3%)	96 (54.9%)		

a Chi-square, b *t*-test. BMI, body mass index; Weekly frequency of SSB/Desserts/WFF, the weekly consumption frequency of sugar-sweetened beverages, desserts, and Western fast food; PSS, The Perceived Stress Scale; EPDS, The self-rating Edinburgh Postnatal Depression Scale; BIPS, The Body Image in Pregnancy; SSRS, The Social Support Rating Scale; PMT, Protective motivation for gestational weight management score; TDEE, Total Daily Energy Expenditure from physical activities; LPA time, Light-intensity physical activity time; MPA time, Moderate-intensity physical activity time; VPA time, Vigorous-intensity physical activity time.

### 3.2 Feature selection for model construction

**Figure 1:**
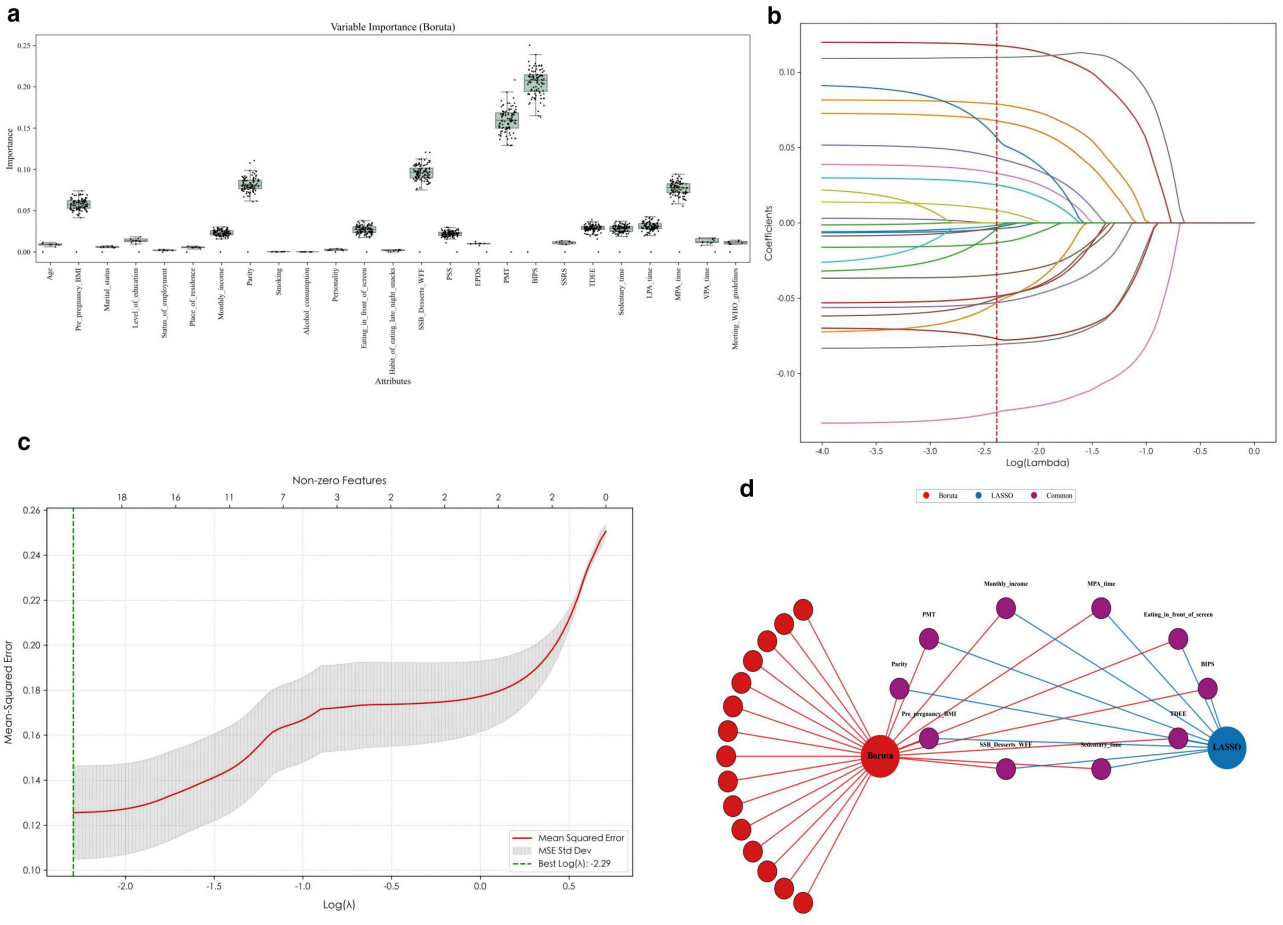
Feature selection results. **(a)** Feature importance scores from the Boruta algorithm. Higher scores indicate greater importance in distinguishing the outcome. All 25 candidate predictors were confirmed as relevant. **(b)** LASSO coefficient paths as a function of log(λ). **(c)** Cross-validation mean squared error (MSE) curve. **(d)** Network diagram showing the overlap of features selected by Boruta and LASSO. The three nodes represent the selection methods (Boruta, LASSO) and their intersection. LASSO, least absolute shrinkage and selection operator; BMI, body mass index; PSS, The Perceived Stress Scale; EPDS, The self-rating Edinburgh Postnatal Depression Scale; BIPS, The Body Image in Pregnancy; SSRS, The Social Support Rating Scale; PMT score, Protective motivation for gestational weight management score; TDEE, Total Daily Energy Expenditure from physical activities; LPA time, Light-intensity physical activity time; MPA time, Moderate-intensity physical activity time; VPA time, Vigorous-intensity physical activity time; SSB/Desserts/WFF, the weekly consumption frequency of sugar-sweetened beverages, desserts, and Western fast food; Meeting WHO guidelines, Meeting WHO guidelines for physical activity.

After data cleaning, the final analytic dataset contained 0% missingness across all 25 candidate predictors; therefore, imputation was not applied in the final modeling dataset. The Boruta algorithm ranked all 25 candidate predictors by importance (Figure 1A), all features were confirmed as relevant based on comparison with shadow features. LASSO regression with 10-fold cross-validation selected the optimal penalty coefficient (λ = 0.004535), which retained 10 predictors with non-zero coefficients, as illustrated by the coefficient paths in Figure 1B. In LASSO cross-validation, the mean squared error (MSE) reached its minimum at the selected penalty best log(λ) = −2.29, mean MSE ≈ 0.12–0.13 (Figure 1C), the final 10 predictors were then defined as the intersection of features selected by LASSO and Boruta (Figure 1D). The Boruta and LASSO identified 10 common features—that is, the Protective Motivation for Gestational Weight Management (PMT) score; BIPS score; parity; total daily energy expenditure (TDEE) from physical activities; moderate-intensity physical activity (MPA) time; sedentary time; weekly consumption frequency of SSBs, desserts, and WFF; monthly income; the behavior of eating in front of a screen; and pre-pregnancy BMI.

### 3.3 Model development and validation

**Figure 2:**
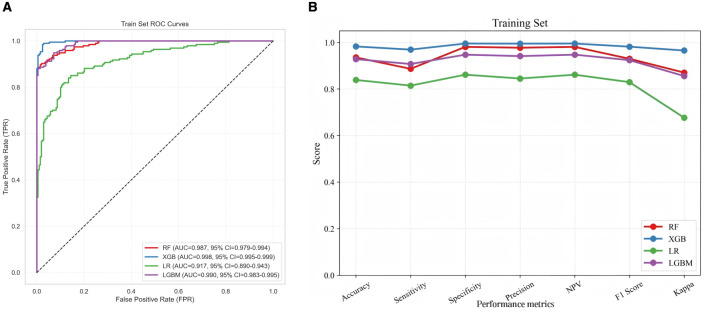
Performance comparison of four machine learning models in the training dataset. **(a)** ROC curves analysis for four machine learning models; **(b)** Evaluation metrics for the training dataset. AUC, area under curve; ROC, receiver operating characteristic; NPV, negative predictive value; RF, random forest; XGB, extreme gradient boosting; LR, logistic regression; LGBM, light gradient boosting machine.

Four models—that is, the RF, XGBoost, LightGBM, and LR models—were trained and validated. The parameter grids were as follows: RF (*n_estimators*: 100/200/300; *max_depth*: 3/5/7; *min_samples_split*: 2/5/10; *min_samples_leaf* : 1/2; 54 combinations), XGBoost (*n_estimators*: 100/200/300; *max_depth*: 3/4/5; *learning_rate*: 0.01/0.05/0.1; *reg_alpha*: 0/0.01/0.1; *reg_lambda*: 0.1/1/10; 243 combinations), LR (*C:* 0.001/0.005/0.01/0.05/0.1; *penalty*: L1/L2; *solver: liblinear*; 10 combinations), and LightGBM (*n_estimators*: 100/200/300; *max_depth*: 3/5/7; *learning_rate*: 0.01/0.03/0.1; *num_leaves*: 7/15; *reg_alpha*: 0.1/0.5; *reg_lambda*: 5/10; 216 combinations). Hyperparameter tuning was performed using GridSearchCV with stratified 5-fold cross-validation, using AUC as the optimization metric. The optimal hyperparameters and corresponding cross-validation performance of each model are summarized in Table 2. After the optimal hyperparameters were determined, the models were trained on the training dataset and their performances were assessed via internal validation and validated externally using an independent test dataset. In the training set, all models demonstrated strong performance, with XGBoost achieving the highest AUC (0.998, 95% CI: 0.995–0.999), followed by LightGBM (0.990, 95% CI: 0.983–0.995). XGBoost also outperformed others across accuracy, sensitivity, specificity, and the Kappa coefficient, while logistic regression (LR) performed least effectively (Figure 2).

**Table 2:** Final hyperparameter settings selected by GridSearchCV and corresponding cross-validation performance.

Model	Final hyperparameter settings	Mean CV AUC ±SD
RF	max_depth = 7, min_samples_leaf = 1, min_samples_split = 5, n_estimators = 200	0.9286 ± 0.0103
XGBoost	learning_rate = 0.1, max_depth = 3, n_estimators = 200, reg_alpha = 0.1, reg_lambda = 10	0.9371 ± 0.0221
LightGBM	learning_rate = 0.05, max_depth = 3, n_estimators = 200, num_leaves = 15, reg_alpha = 0.5, reg_lambda = 5	0.9280 ± 0.0152
LR	C = 0.01, penalty = L2, solver = liblinear	0.9063 ± 0.0152

RF, random forest; LR, logistic regression; CV, cross-validation; AUC, area under the receiver operating characteristic curve; SD, standard deviation. Hyperparameters were selected using GridSearchCV and used for final model training. Values are presented as mean CV AUC ± SD.

**Figure 3:**
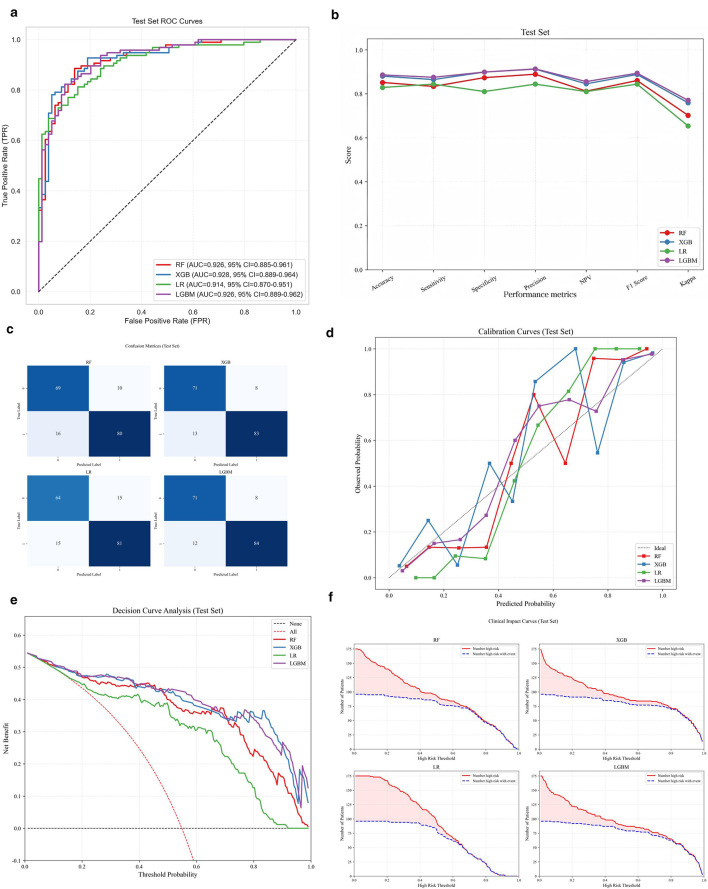
Performance comparison of four machine learning models in the test dataset. **(a)** ROC curve analysis; **(b)** Evaluation metrics for the test dataset; **(C)** Confusion matrices. Rows represent true labels, columns represent predicted labels. Diagonal cells indicate correct classifications, and off-diagonal cells indicate misclassifications. Color intensity reflects sample count; **(d)** Calibration plots. Calibration plots showing the agreement between predicted and observed event probabilities. The dashed diagonal line indicates perfect calibration; curves closer to this line indicate better calibration; **(e)** Decision curves. The x-axis represents the threshold probability, and the y-axis represents the net benefit. The “treat all” and “treat none strategies are shown as reference lines. Higher curves indicate greater clinical utility across the corresponding threshold range; **(f)** Clinical impact curves. The x-axis represents the high-risk threshold probability, and the y-axis represents, per 1,000 individuals, the number classified as high risk (dashed line) and the number of true positives (solid line). A smaller gap between the two curves indicates fewer false positives and better classification performance. AUC, area under curve; ROC, receiver operating characteristic; NPV, negative predictive value; RF, random forest; XGB, extreme gradient boosting; LR, logistic regression; LGBM, light gradient boosting machine.

In the test set, receiver operating characteristic (ROC) curves for four models as shown in Figure 3A, XGBoost showed a slightly higher AUC (0.928, 95% CI: 0.889–0.964) than LightGBM (0.926, 95% CI: 0.889–0.962). Pairwise comparisons of the AUC values for all four models were then conducted using the DeLong non-parametric test, The results (Table 3) indicated that the AUC differences between any two models were not statistically significant (*P* > 0.05). The detailed performance metrics of the four models on the test set are summarized in Figure 3B. LightGBM achieved the highest sensitivity, specificity, precision, F1-score, Kappa coefficient. Confusion matrix analysis (Figure 3C) revealed that LightGBM had the highest sensitivity (87.5%) and accuracy (88.6%), precision (91.3%), with 71 true negatives, 8 false positives, 12 false negatives, and 84 true positives. XGBoost followed closely but had more false negatives (*FN* = 13), resulting in slightly lower sensitivity (86.5%). Random forest (RF) demonstrated moderate performance (accuracy = 85.1%), while LR had the lowest specificity and accuracy (82.9%). To assess the stability of performance in the test cohort, we reported bootstrap 95% confidence intervals for the main classification metrics (sensitivity, specificity, accuracy, precision, F1-score, and Kappa coefficient) across the four models (Table 4). Calibration curves (Figure 3D) showed that LightGBM and XGBoost were closest to the ideal calibration line, indicating the best alignment between predicted probabilities and actual EGWG incidence. Decision curve analysis (DCA) (Figure 3E) confirmed that both models provided the highest net clinical benefit across all threshold ranges (0–1.0), with LightGBM slightly surpassing XGBoost in the mid-to-high threshold range (0.4–0.8). Clinical impact curves (Figure 3F) further confirmed stable high-risk identification by LightGBM and XGBoost, with LightGBM showing slightly better net benefit in the clinically relevant threshold range (0.2–0.7). Based on performance in the test dataset, LightGBM was identified as the optimal model for EGWG risk prediction, with XGBoost as a close second.

**Table 3:** DeLong test results for pairwise comparisons of AUCs among four machine learning models in the test cohort.

Comparison	AUC (new model)	AUC (reference model)	ΔAUC	95% CI for ΔAUC (1,000 bootstrap resamples)	Z statistic	*P-*value
XGB vs. RF	0.928	0.926	0.002	−0.031~0.035	0.112	0.9108
LR vs. RF	0.914	0.926	−0.012	−0.052~0.028	−0.589	0.5559
LGBM vs. RF	0.926	0.926	0.000	−0.033~0.033	0.000	1.0000
LR vs. XGB	0.914	0.928	−0.014	−0.054~0.026	−0.691	0.4895
LGBM vs. XGB	0.926	0.928	−0.002	−0.035~0.031	−0.112	0.9108
LGBM vs LR	0.926	0.914	0.012	−0.028~0.052	0.589	0.5559

AUC, area under curve; RF, random forest; XGB, extreme gradient boosting; LR, logistic regression; LGBM, light gradient boosting machine; CI, confidence interval. Pairwise AUC comparisons were performed using the DeLong test. The 95% CIs for ΔAUC were estimated by bootstrap with 1,000 resamples. ΔAUC was calculated as AUC (new model) – AUC (reference model). A two-sided *P* value < 0.05 was considered statistically significant.

**Table 4:** Classification performance of four machine learning models in the test cohort with bootstrap 95% confidence intervals.

Model	Sensitivity (95% CI)	Specificity (95% CI)	Accuracy (95% CI)	Precision (95% CI)	F1-score (95% CI)	kappa (95% CI)
RF	0.833 (0.749–0.904)	0.873 (0.797–0.937)	0.851 (0.789–0.903)	0.889 (0.820–0.944)	0.860 (0.805–0.908)	0.702 (0.590–0.795)
XGB	0.865 (0.792–0.927)	0.899 (0.835–0.962)	0.880 (0.829–0.931)	0.912 (0.857–0.962)	0.888 (0.837–0.930)	0.759 (0.658–0.849)
LR	0.844 (0.771–0.912)	0.810 (0.709–0.899)	0.829 (0.760–0.891)	0.844 (0.770–0.909)	0.844 (0.785–0.895)	0.654 (0.540–0.759)
GBM	0.875 (0.800–0.938)	0.899 (0.835–0.962)	0.886 (0.834–0.931)	0.913 (0.861–0.962)	0.894 (0.847–0.935)	0.770 (0.676–0.854)

Values are point estimates with bootstrap-derived 95% confidence intervals (1,000 resamples) in the test cohort. CI, confidence interval; kappa, Cohen's kappa coefficient; RF, random forest; XGB, extreme gradient boosting; LR, logistic regression; LGBM, light gradient boosting machine.

### 3.4 Model interpretability

**Figure 4:**
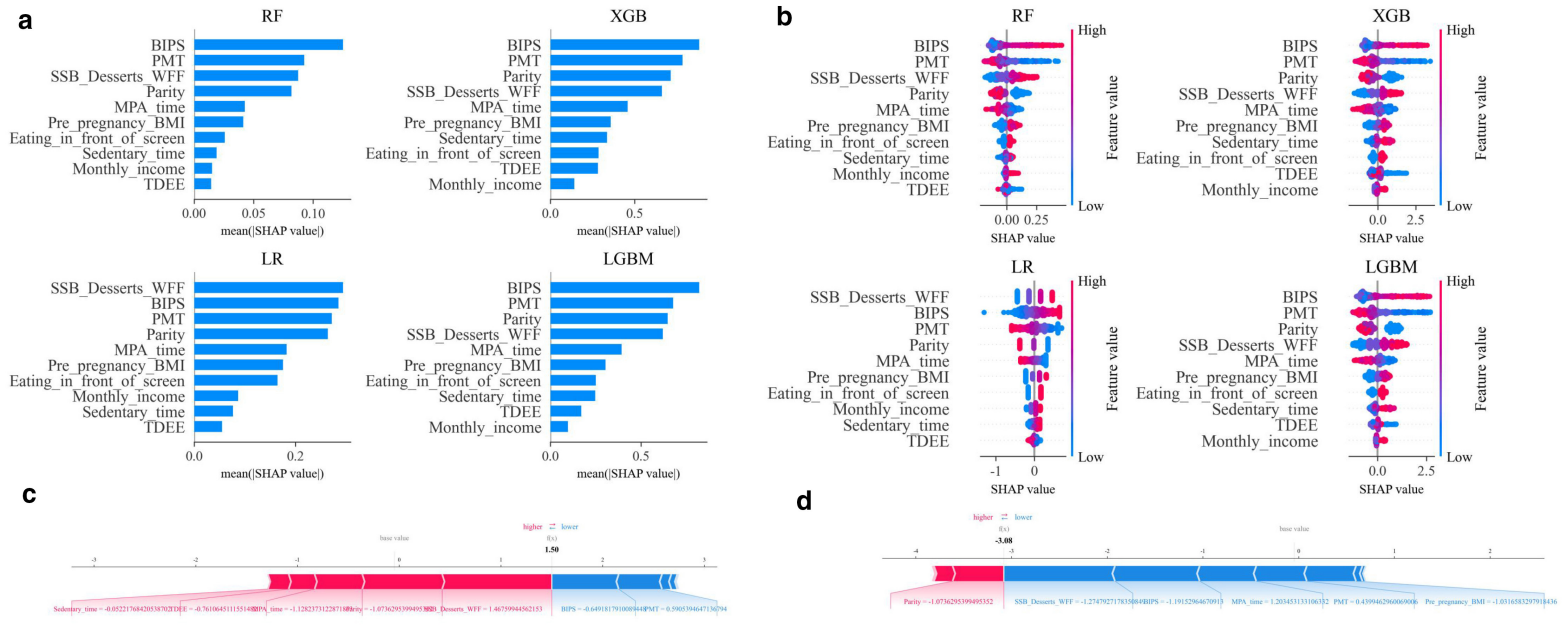
Interpretability analysis of four models. **(a)** Feature importance ranking of four machine learning models; **(b)** SHAP summary bar plot of four machine learning models; Every dot in a row symbolizes a patient, and its color denotes the feature value—red denotes a value that is greater and blue denotes a value that is lower. The more dispersed the points of the graph represent the greater the impact of the variables on the model. **(c, d)** Individualized SHAP force plots for two representative participants on the LightGBM models, Red indicates features that increased the model output (higher predicted risk), whereas blue indicates features that decreased the model output (lower predicted risk). The gray line represents the base value (average model output). Higher functional significance is indicated by longer bars. These plots show how individual features contributed to the prediction for each sample. SHAP, SHapley Additive explanation; BIPS, The Body Image in Pregnancy; PMT, Protective motivation for gestational weight management score; SSB/Desserts/WFF, the weekly consumption frequency of sugar-sweetened beverages, desserts, and Western fast food; TDEE, Total Daily Energy Expenditure from physical activities; MPA time, Moderate-intensity physical activity time; BMI, body mass index.

This study employed the SHAP framework to conduct an interpretability analysis of four models, evaluating the consistency of key feature importance across different modeling strategies. The SHAP feature importance bar chart (Figure 4A) reveals that among the top ten key features, the five most influential ones are: BIPS, PMT, parity, weekly consumption frequency of SSBs, desserts, and WFF, and MPA time. The SHAP feature importance rankings across the four models show a high degree of consistency, especially the top five key features, with the dominance of these core predictive factors being stable across different algorithms.

The SHAP summary plot (Figure 4B) further confirms that the effect direction of each key feature on EGWG risk is consistent across four models. Specifically, higher values in PMT, parity, MPA time, and TDEE (red points) correspond to lower SHAP values, indicating a reduced risk of EGWG, and thus, acting as protective factors. Conversely, higher values for the BIPS, weekly consumption frequency of SSBs, desserts, and WFF, pre-pregnancy BMI, sedentary time, the behavior of eating in front of a screen, and monthly income (red points) are associated with higher SHAP values, suggesting an increased risk of EGWG, thus functioning as risk factors.

To better understand the decision-making process of the model at the individual level, an interpretative analysis of two representative cases was performed, as shown in Figures 4C, D. For the representative high-risk participant (Figure 4C), the model output increased to *f* (x) = 1.50, mainly driven by weekly consumption frequency of SSBs, desserts, and WFF, parity, MPA_time, TDEE, and sedentary time. For the representative low-risk participant (Figure 4D), the model output decreased to *f* (x) = −3.08, primarily driven by pre-pregnancy BMI, PMT, MPA_time, BIPS, and weekly consumption frequency of SSBs, desserts, and WFF. Overall, the predictions were jointly determined by multiple features, supporting the interpretability of the model at the individual level.

### 3.5 Implementation of the web calculator

**Figure 5:**
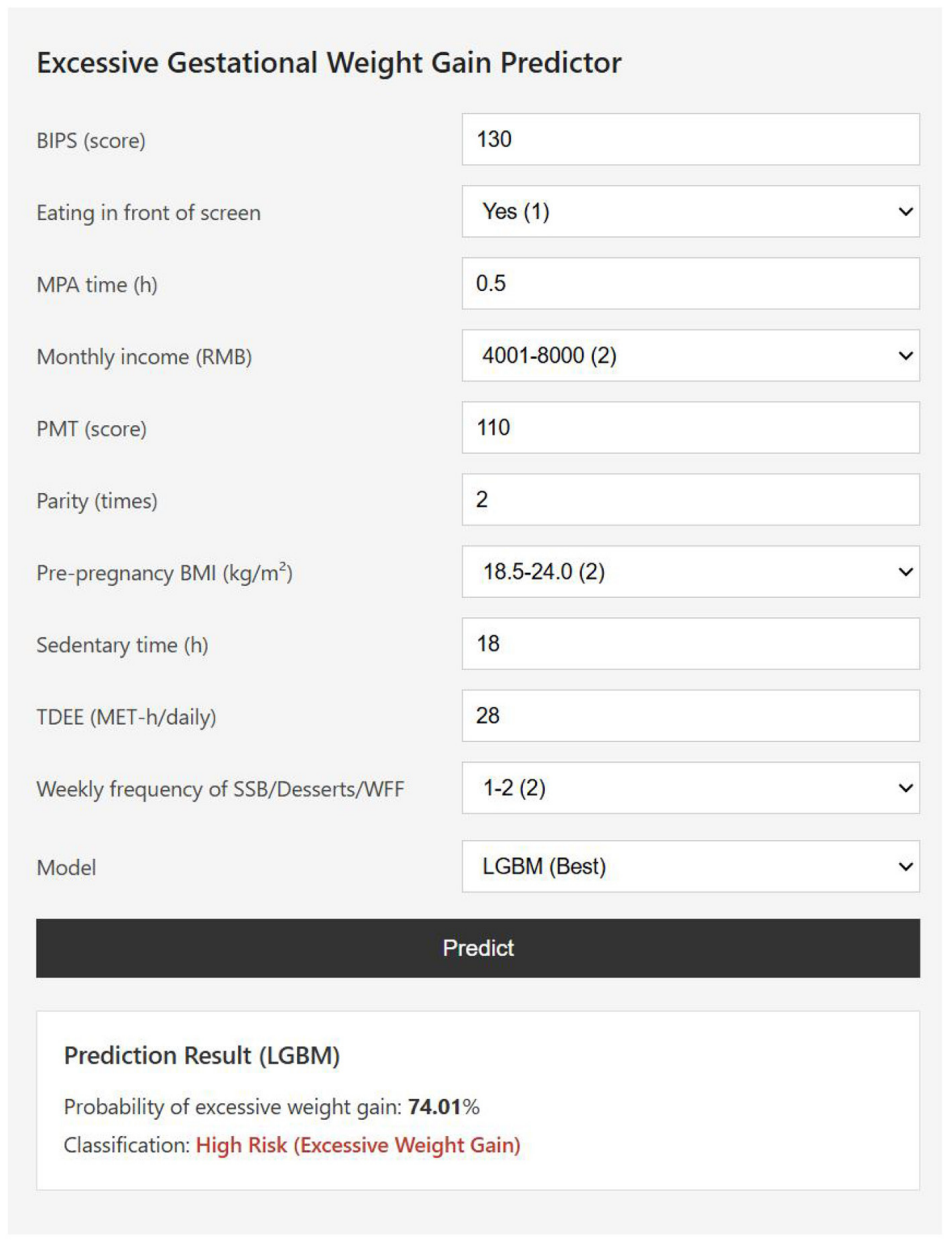
The web-based calculator for predicting excessive gestational weight gain. By simply inputting all ten predictors information: the body image in pregnancy scale score (BIPS), protective motivation for gestational weight management score (PMT), parity, the weekly consumption frequency of sugar-sweetened beverages, desserts, and Western fast food (SSB/Desserts/WFF), moderate-intensity physical activity time (MPA time), eating in front of screen, total daily energy expenditure from physical activities (TDEE), pre-pregnancy body mass index(BMI), Sedentary time, monthly income, it is possible to predict the risk of excessive gestational weight gain. BIPS, The Body Image in Pregnancy; PMT score, Protective motivation for gestational weight management score; Weekly frequency of SSB/Desserts/WFF, the weekly consumption frequency of sugar-sweetened beverages, desserts, and Western fast food; TDEE, Total Daily Energy Expenditure from physical activities; LPA time, Light-intensity physical activity time; MPA time, Moderate-intensity physical activity time; BMI, body mass index.

To facilitate the clinical application of the predictive models, a web-based interactive calculator was developed using the Python programming language (version 3.14.3). The web application was built with the Flask micro-framework, and derived from the final machine-learning prediction model. The application runs locally and can be accessed via a web browser, providing an intuitive interface for clinicians to estimate individual patient risk. Users enter the 10 prespecified predictors, and the tool returns a real-time predicted probability of EGWG. For example (Figure 5), after the required variables are entered, the calculator outputs an estimated EGWG probability of 74.01% for the illustrated participant. The web calculator is accessible at: http://39.103.64.176/.

## 4 Discussion

This research developed a ML prediction model and identified essential factors for predicting EGWG. The findings offered a useful tool for decision-making in obstetric risk assessment and management by employing feature-selection techniques and multiple model validation.

Among the four machine learning models, LightGBM was considered the preferred model because it showed a more favorable threshold-dependent performance profile, more stable calibration, and greater clinical utility in the test dataset. Although XGBoost yielded a slightly higher AUC than LightGBM, the DeLong pairwise comparisons indicated that the differences in AUC between models were not statistically significant. Therefore, model selection should not be based on AUROC alone, but should place greater emphasis on threshold-dependent metrics that are more directly relevant to clinical screening, such as sensitivity, specificity, false-negative burden, and overall classification performance (27). Within this context, the consistent advantage of LightGBM in sensitivity, F1-score, and Kappa coefficient in the test dataset appears clinically meaningful. For early screening of EGWG, minimizing false negatives is particularly important, as failure to identify high-risk pregnant women may delay individualized counseling and targeted lifestyle interventions. Meanwhile, the comparable specificity of LightGBM and XGBoost suggests that the improved sensitivity of LightGBM was not achieved at the cost of a substantial increase in false-positive classifications. Accordingly, the advantage of LightGBM should be interpreted as reflecting a more favorable balance of classification performance at the operating threshold, rather than a marked superiority in overall discrimination.

The calibration and decision-analytic findings further support this interpretation. Both LightGBM and XGBoost showed good calibration, indicating that their predicted probabilities were generally consistent with the observed risk of EGWG. However, the slightly more stable calibration profile of LightGBM suggests that it may provide more reliable estimates for individualized risk assessment and risk-stratified intervention (28). In addition, LightGBM maintained a slight advantage within the key threshold intervals. This pattern suggests that LightGBM may offer a more favorable balance between early identification of high-risk pregnancies and avoidance of unnecessary high-risk labeling (29). These findings have clear clinical implications. Previous studies have shown that EGWG is associated with multiple adverse maternal and neonatal outcomes, highlighting the importance of identifying high-risk women early in pregnancy for improved antenatal management (30). Prior studies have also demonstrated that early prediction of excessive gestational weight gain is feasible when demographic, behavioral, and psychosocial factors are considered jointly (9, 10). Building on this evidence, the present study further suggests that an interpretable machine-learning framework can not only maintain good performance when tested on a temporally distinct dataset, but also support individualized risk explanation, thereby informing more targeted guidance on diet, physical activity, sedentary behavior, and weight management.

Another strength of this study is that SHAP analysis was performed for all four models rather than being limited to the final selected model. The high consistency of the top-ranked predictors across algorithms supports the robustness of the identified risk structure, while the concordant effect directions of the major features further increase confidence that these findings were not artifacts of a single modeling strategy. Specifically, stronger pregnancy weight-management motivation, higher parity, longer PMA time, and higher total daily energy expenditure were associated with lower predicted EGWG risk. In contrast, greater body image concern, more frequent unhealthy dietary intake, higher pre-pregnancy BMI, longer sedentary time, screen-related eating behavior, and higher monthly income were associated with increased predicted risk. These findings suggest that EGWG is shaped by an interplay of demographic, behavioral, and psychosocial factors, some of which may be modifiable (10, 31).

Psychological factors—such as body image and protective motivation—are important predictors of weight control during pregnancy. These factors affect weight-management behavior. This finding is consistent with that reported by J-P et al. (32). Their study showed that women with poor body image often had negative views on weight gain. These women may have ignored weight management. They may also have adopted unhealthy dietary practices and reduced physical activity, increasing the risk of EGWG. Rodgers et al. (33) reported similar results. Their study showed that that individuals with poor body image may be more vulnerable to sociocultural pressures regarding ideal body standards, which can heighten psychological stress and negatively impact weight regulation behaviors. The Protection Motivation Theory explains these patterns. Differences in weight management behavior may be due to differences in risk awareness and coping abilities. Pregnant women with low protective motivation may underestimate the risk of EGWG. They may also show limited intention to take corrective action, a finding consistent with those of Ge et al. (34). Peng et al. (35) also reported related results. Their study showed that low protective motivation could be linked to low self-efficacy, reducing their participation in health promoting behaviors. These findings emphasize on the need to address the psychological and motivational factors in maternal weight control. Furthermore, it is suggested that more research on the psychological and motivational behavioral mechanism for weight management among pregnant women should be done, with the purpose to improve bodyweight cognition based on internal motivation and main psychosocial determinants, increasing self-efficacy, and finally resulting in sustained healthy behavior.

In the SHAP analysis, parity is a significant predictor of EGWG from a social perspective. Based on the domestic and international evidence (3, 10), multiparous women have a lower risk of EGWG than primiparous women, as multiparous women may have more experience with weight management and stronger self-regulation abilities. Higher monthly income has also been positively associated with EGWG. According to Adeoye et al. (36), women with higher incomes had more access to calorie-dense foods and less time for physical activity because of their job demands. Similarly, this group was likely to eat convenience foods of high caloric density and subpar nutritional value.

Weekly frequency of consuming sugar-sweetened beverages, desserts, and Western-style fast food is also an important predictor of EGWG, which is consistent with evidence that diets high in sugar, fat, and ultra-processed foods can lead to excess weight gain (37). Eating in front of a screen while watching TV or using a phone while eating was identified as a risk factor, which is consistent with the work of McDonald et al. (10). This behavior takes attention away from eating, delays feeling full, and makes eating last longer, finally leads to increased calorie intake and raises the risk of EGWG (10).

Total daily energy expenditure is a critical factor in weight management, which is the sum of the resting metabolic rate (RMR), diet-induced thermogenesis and physical activity expenditure. While Although rest metabolism adapts to physiological changes throughout pregnancy, physical activity continues to be an important determining factor (38). Lower EGWG risk appears to be associated with higher physical activity expenditure and longer moderate-intensity activity duration; for example, MPA increases energy expenditure, increases insulin sensitivity, maintains glucose metabolism, and reduces excess weight gain (39). According to international guidelines from the WHO, UK, and Canada, pregnant women are advised to engage in a minimum of 150 min of MPA weekly (39). The risk of EGWG is raised considerably by prolonged sedentary behavior.A positive energy balance results from a lower metabolic rate and overall energy expenditure as a consequence of sedentary behavior, it also promotes visceral fat accumulation, and is related to glucose variability and insulin sensitivity, eventually triggering metabolic dysfunction (40, 41). A 2025 American Heart Association (AHA) review identified a link between sedentary behavior during pregnancy and factors such as preterm birth, changes in maternal blood pressure, lipid levels, glucose, and GWG (42). These findings proved that sedentary behavior affected both pregnancy outcomes and GWG. Further research is required to evaluate the relationship between different levels of sedentary time and weight gain, and to develop evidence-based recommendations for decreasing sedentary time during pregnancy.

These findings have major clinical implications for clinical practice. First, they underscore the potential value of reducing screen time and interrupting prolonged sedentary behavior as strategies for gestational weight management. Second, precise energy balance management should be provided to pregnant women with individualized recommendations for caloric intake based on prepregnancy BMI and levels of MPA. Finally, pregnant women should be encouraged to practice mindful eating, create screen-free eating environments, and maintain regular physical activity, accumulating at least 150 min of MPA per week.

There are several limitations in this study. First, this was a single-center study conducted in one tertiary hospital, which may limit representativeness and restrict generalizability to broader Chinese or international populations with different sociodemographic characteristics, care pathways, or measurement practices. Second, although we evaluated model performance in an independent cohort, the test dataset originated from the same institution and therefore represents a temporal test split rather than geographic external validation. Testing on a temporally distinct dataset provides evidence of stability and reproducibility over time and reduces the risk of overfitting to a single recruitment period; however, it does not fully assess transportability across different hospitals, regions, or health-care systems. Moreover, the test cohort was modest in size, which may introduce sampling variability in performance estimates—particularly for threshold-dependent metrics derived from the confusion matrix. Accordingly, performance differences between models should be interpreted cautiously, and we reported bootstrap-based confidence intervals for key classification metrics to better characterize uncertainty. Third, several predictors (e.g., dietary behaviors and physical activity) were derived from self-reported questionnaires and may be subject to recall and reporting bias. To reduce this risk, we used validated instruments (e.g., PPAQ) and standardized on-site administration with immediate quality checks for omissions and logical inconsistencies; nevertheless, residual measurement error cannot be excluded.

To strengthen clinical translation, future work will focus on improving model transportability and measurement validity. Specifically, we will conduct multicenter, multi-region geographic external validation (e.g., across different provinces and urban–rural settings) to evaluate generalizability across heterogeneous populations and care environments. Where multicenter data are available, we will consider internal–external cross-validation frameworks to quantify between-site performance variability and improve robustness. In addition, we will incorporate more objective or hybrid measurements to reduce self-report bias, including accelerometer- or wearable-derived physical activity and sedentary time, structured dietary assessment (e.g., repeated 24-h dietary recalls, or digital dietary records), and relevant metabolic biomarkers. Moreover, questionnaire administration began at 14 gestational weeks and was completed on average at 18–19 weeks (early second trimester). Our literature review suggests this timing coincides with the early phase of accelerated gestational weight gain, leaving an adequate window for intervention and trajectory modification (43, 44). Nonetheless, moving screening to the first prenatal visit or earlier pregnancy may further optimize timeliness; future studies will assess the feasibility and its potential to enhance intervention effectiveness, with the aim of informing more precise timing recommendations for gestational weight management. Although the initial sample size planning considered a broader set of candidate predictors, the final model included only 10 predictors after feature selection, resulting in a favorable events-per-predictor and supporting stable model estimation.

## 5 Conclusions

In this prospective observational study, we developed and temporally validated an interpretable ML framework for the early prediction of EGWG in Chinese pregnant women. Using Boruta and LASSO for feature selection, we identified ten key predictors across psychosocial, behavioral, and demographic domains. SHAP analysis showed that pregnancy body image, protective motivation for gestational weight management, parity, weekly consumption of sugar-sweetened beverages, desserts, and Western-style fast food, and moderate-intensity physical activity were the most influential contributors to EGWG risk. Among the four models evaluated, LightGBM demonstrated the best predictive performance in the test cohort. The online calculator further improve clinical usability by enabling rapid, intuitive risk stratification in practice.

Future studies should validate this model in broader Chinese populations across different geographic regions, ethnic groups, and socioeconomic backgrounds to strengthen its generalizability. Further work should also assess its integration into digital clinical workflows, including electronic health record systems, and test whether model-guided interventions can reduce EGWG and improve maternal and child health outcomes. Overall, this interpretable LightGBM-based model provides a robust and clinically actionable tool for the early identification and personalized management of EGWG risk, with the potential to improve prenatal care.

## Data Availability

The raw data supporting the conclusions of this article will be made available by the authors, without undue reservation.
