# Supplementary figures and images for: Interpretable machine-learning model to accurately identify women at risk of excessive gestational weight gain
Source: Front Public Health. 2026 Apr 20;14:1779962. doi: 10.3389/fpubh.2026.1779962 (PMC13136279; doi:10.3389/fpubh.2026.1779962)

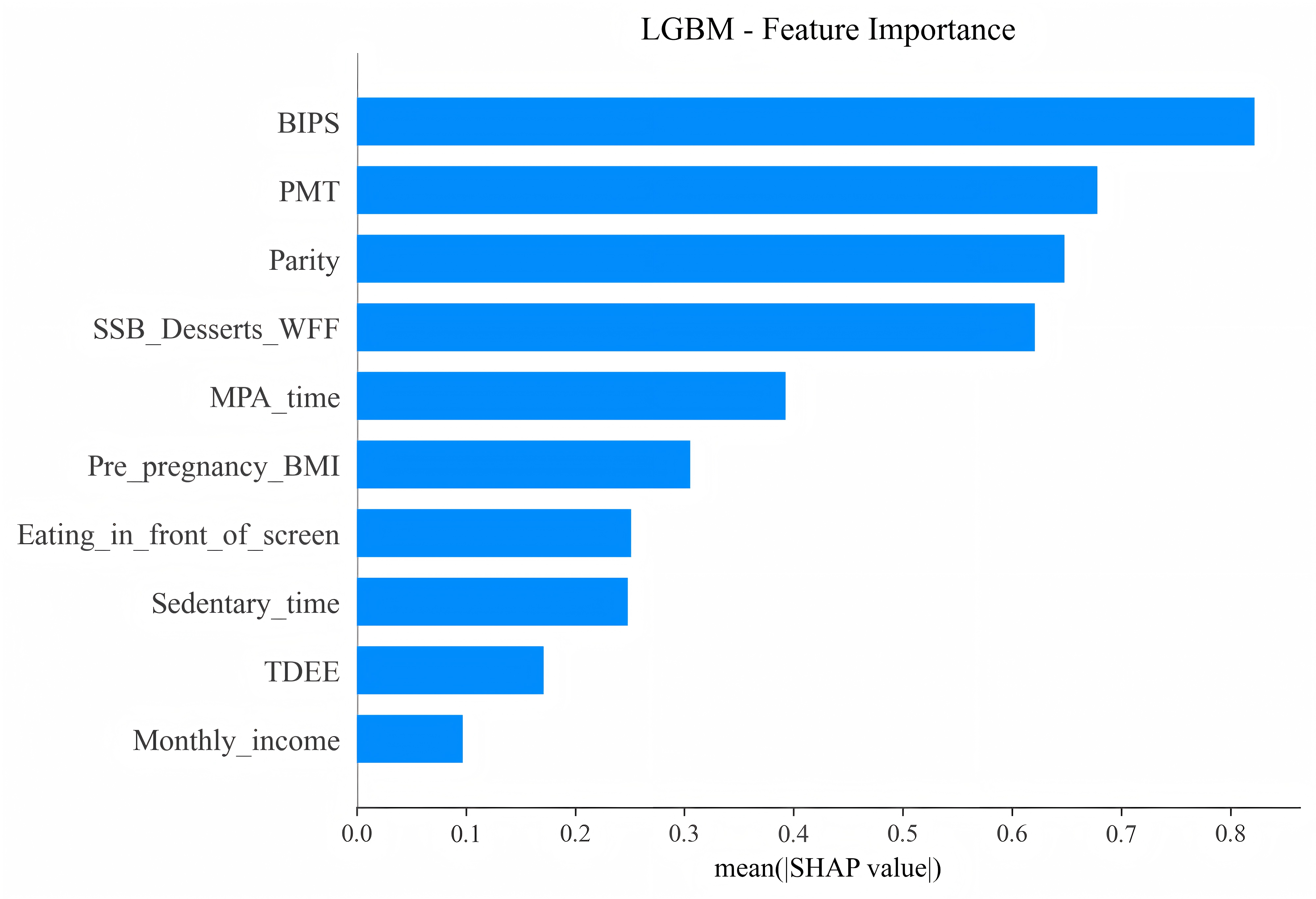

Supplement: Supplementary file 1 [file Image_1.jpeg]

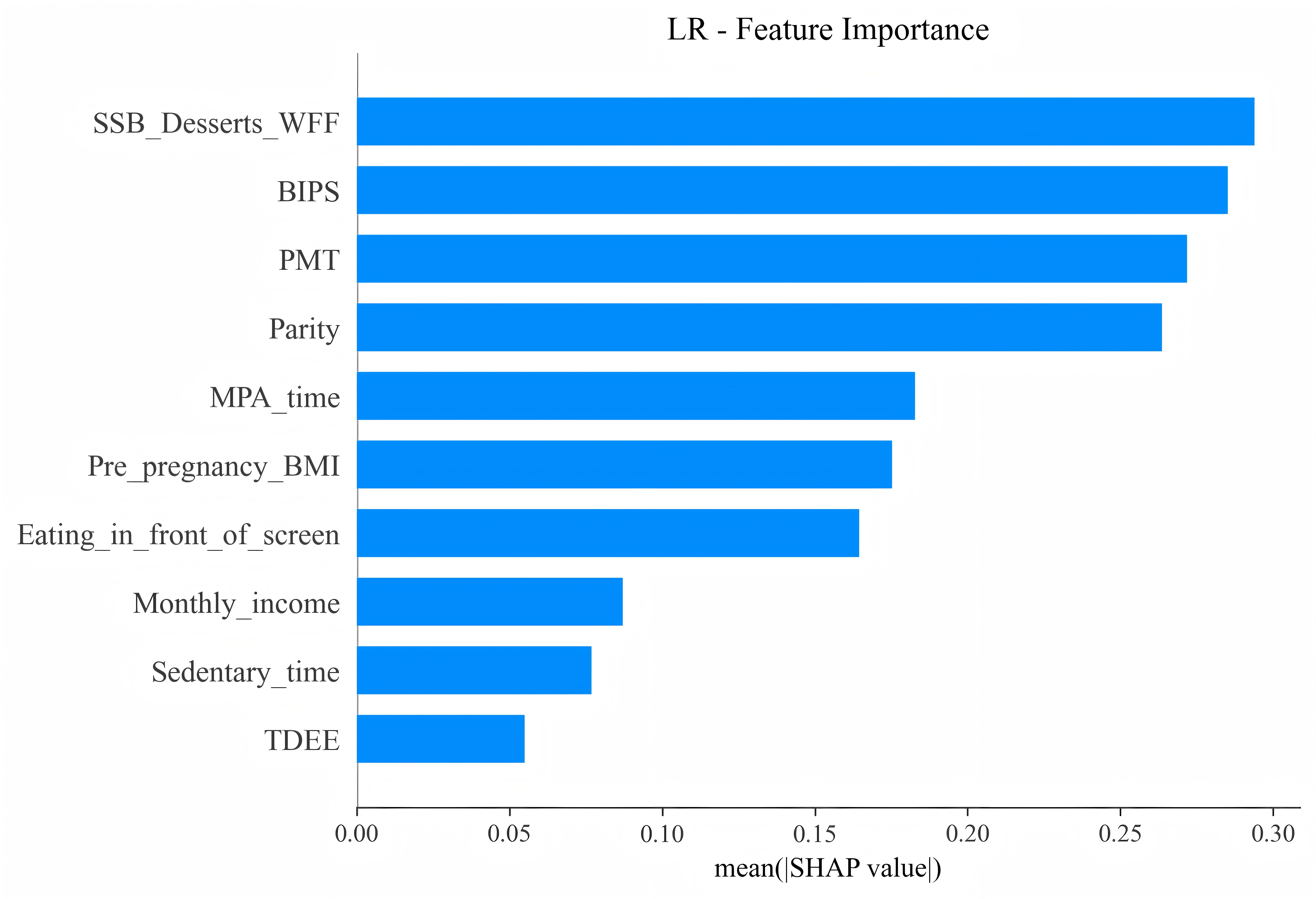

Supplement: Supplementary file 2 [file Image_2.jpeg]

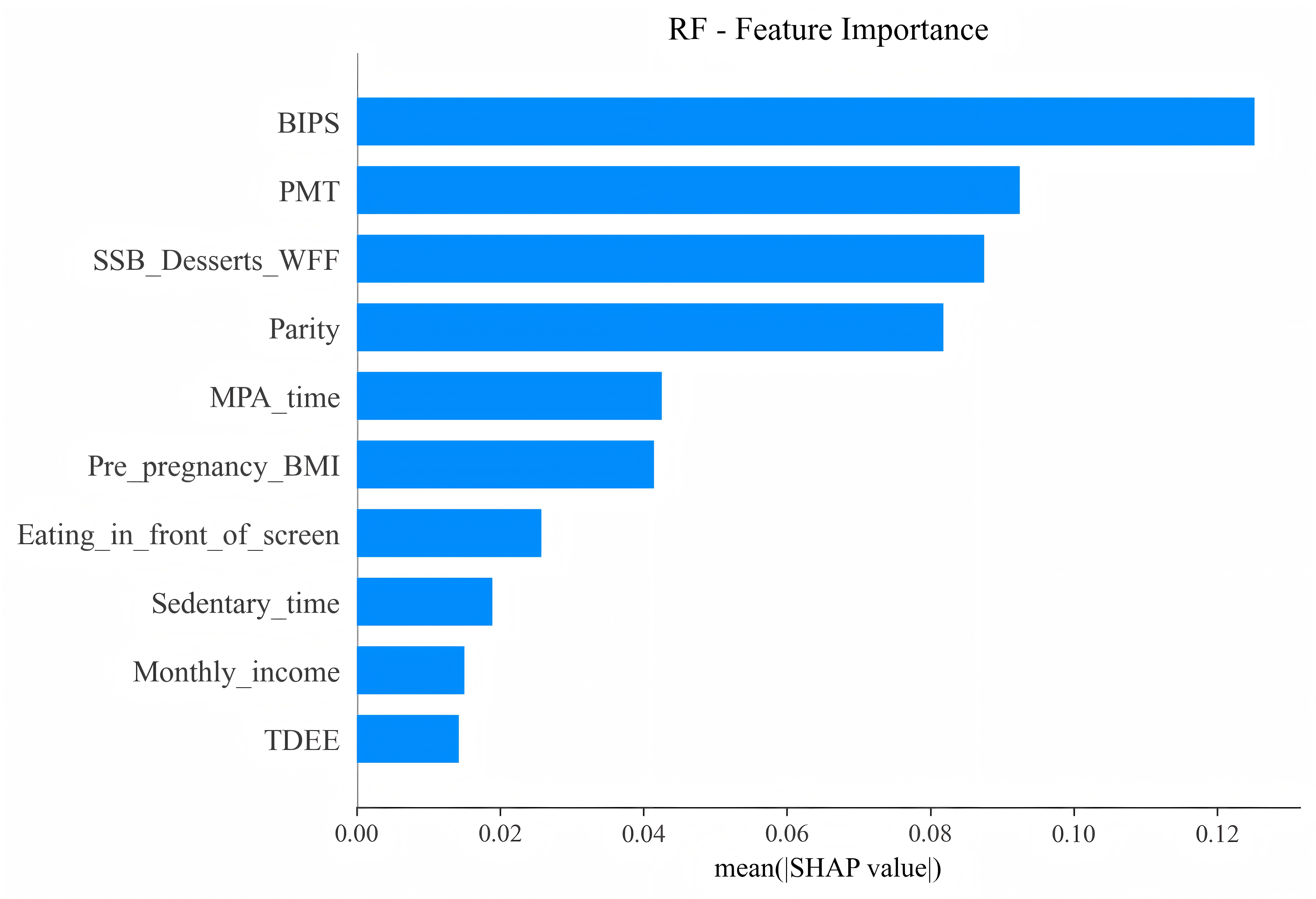

Supplement: Supplementary file 3 [file Image_3.jpeg]

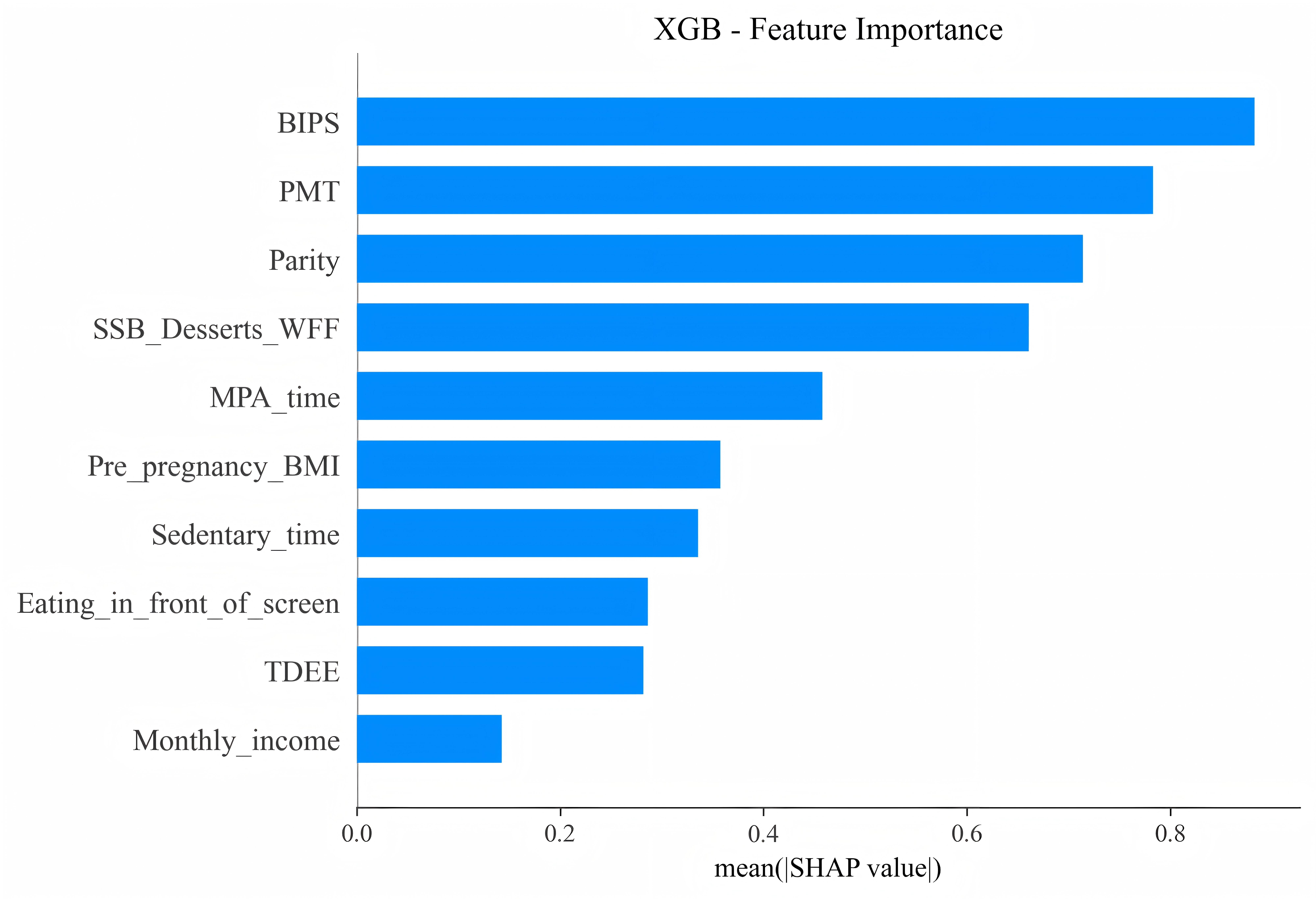

Supplement: Supplementary file 4 [file Image_4.jpeg]

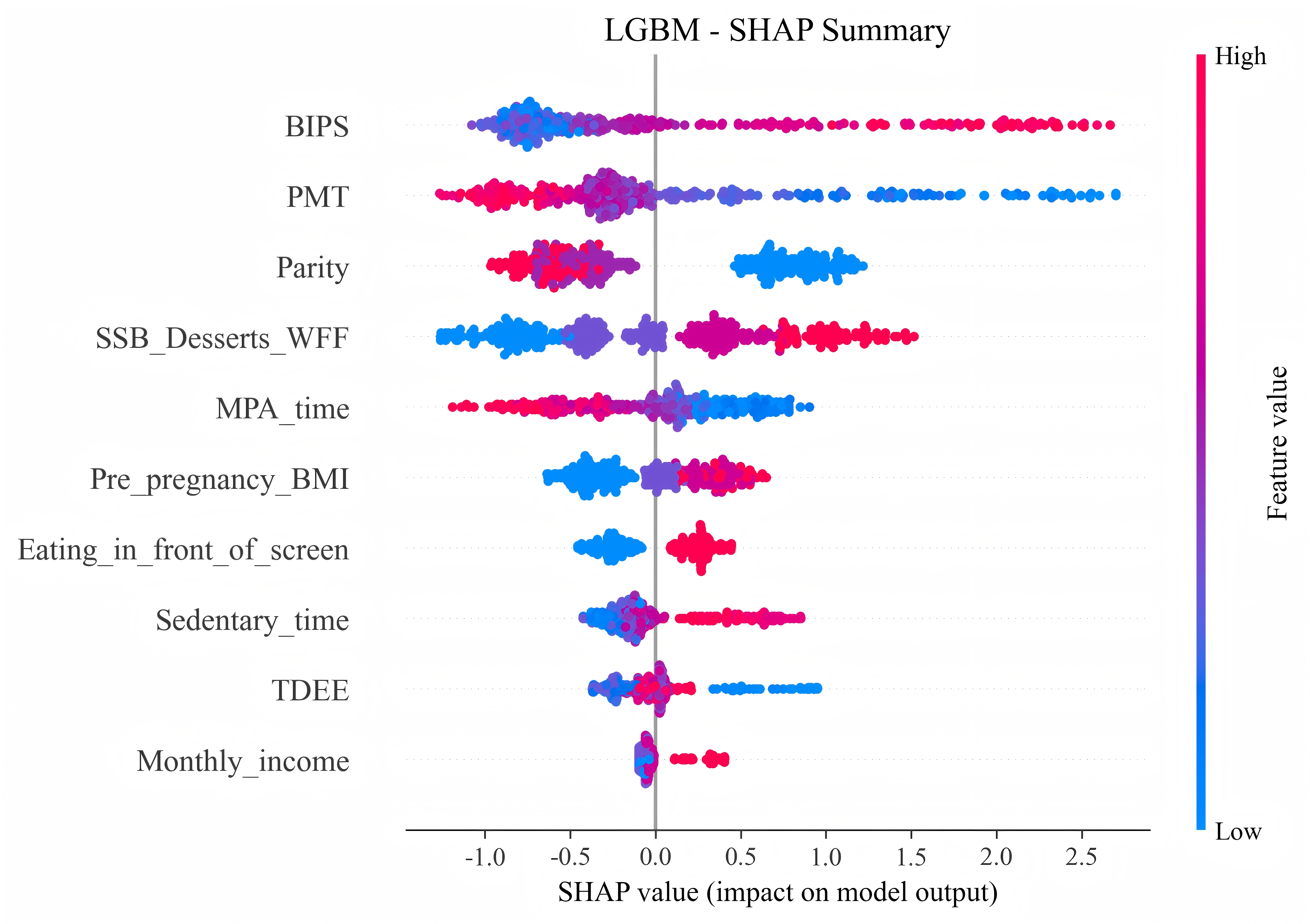

Supplement: Supplementary file 5 [file Image_5.jpeg]

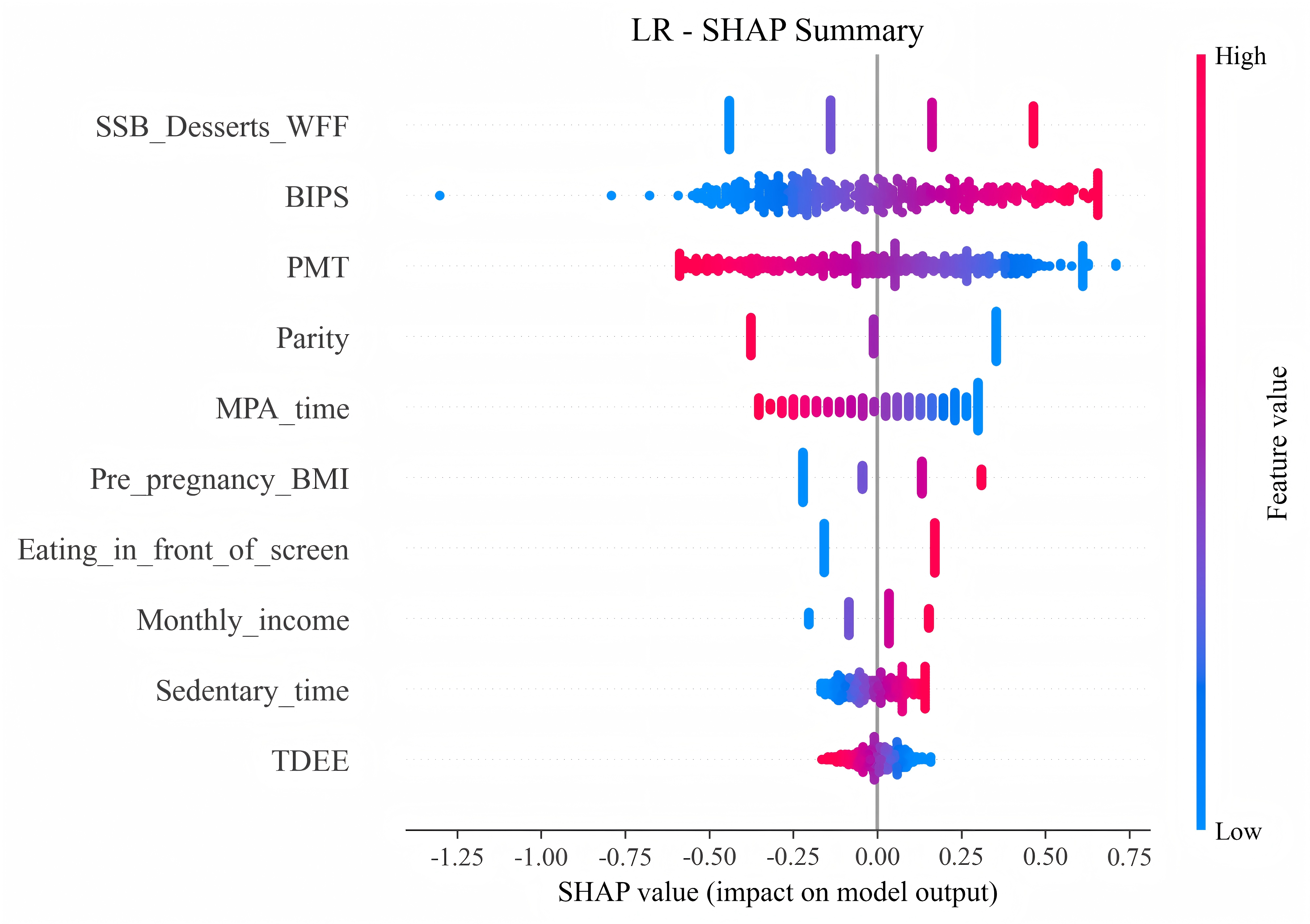

Supplement: Supplementary file 6 [file Image_6.jpeg]

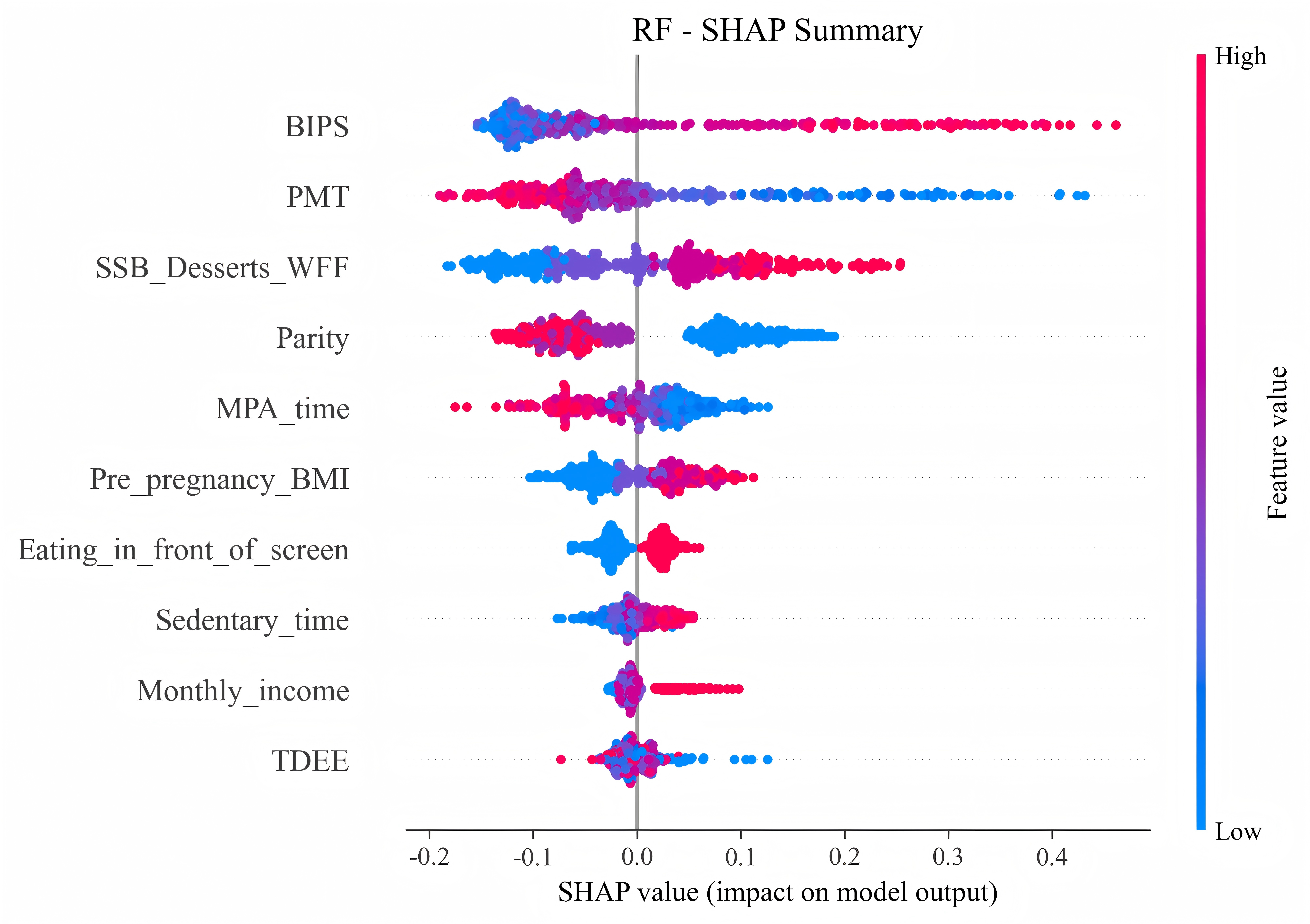

Supplement: Supplementary file 7 [file Image_7.jpeg]

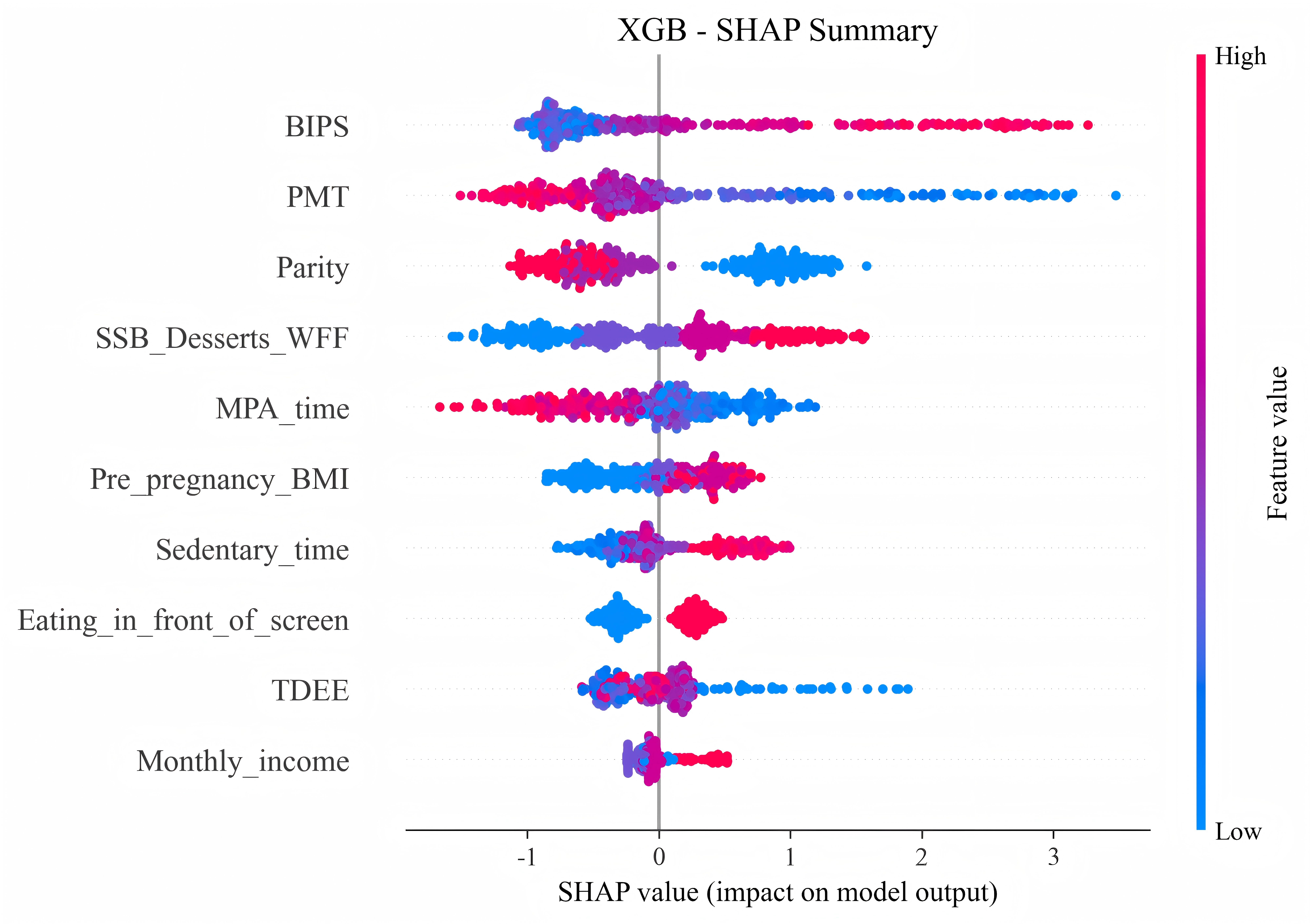

Supplement: Supplementary file 8 [file Image_8.jpeg]
